# Nitrogen along the Hydrological Gradient of Marsh Sediments in a Subtropical Estuary: Pools, Processes, and Fluxes

**DOI:** 10.3390/ijerph16112043

**Published:** 2019-06-09

**Authors:** Weifang Hu, Wenlong Zhang, Linhai Zhang, Chuan Tong, Zhigao Sun, Yuehmin Chen, Congsheng Zeng

**Affiliations:** 1 State Key Laboratory for Subtropical Mountain Ecology of the Ministry of Science and Technology and Fujian Province, Fujian Normal University, Fuzhou 350007, China; weifanghyx@163.com (W.H.); zhangwenlong027@163.com (W.Z.); mary12maryzhang@126.com (L.Z.); tongch@fjnu.edu.cn (C.T.); zhigaosun@163.com (Z.S.); 2 College of Geographical Science, Fujian Normal University, Fuzhou 350007, China

**Keywords:** nitrogen pools, nitrogen processes, nitrogen fluxes, sediments, hydrological gradient

## Abstract

Knowledge on the distribution of nitrogen (N) pools, processes, and fluxes along hydrological gradients provides a comprehensive perspective to understand the underlying causal mechanisms in intertidal flats, and thus improve predictions and climate adaptation strategies. We used a space-for-time substitution method to quantify N pools, processes, and fluxes along a hydrological gradient. Further, we linked N pools and processes and investigated not only surface but also subsurface sediments. Our results showed a gradual decrease in total N (TN) and mineralization rates (*PN_min_*), but an increase in potential rates of nitrification (*PNR*) and denitrification (*PDNR*) under an elevated hydrological gradient, except for TN and *PN_min_* in the subsurface sediment, which accumulated on the interaction zone between the high and middle tidal flats. Most sedimentary ammonium N (NH_4_^+^) and nitrate N (NO_3_^−^) concentrations were similar; however, NH_4_^+^ accumulated on the subsurface of the middle tidal flat. NO_3_^−^ fluxes (from −0.54 to −0.35 mmol m^−2^ h^−1^) were uptake fluxes in the intertidal flats, but NH_4_^+^ fluxes (−2.48–3.54 mmol m^−2^ h^−1^) changed from uptake to efflux in the seaward direction. Structural equation modeling of the effects of inundation frequency, underground biomass, total carbon (TC), electrical conductivity (EC), and clay proportion on the N processes revealed that these accounted for 67%, 82%, and 17% of the variance of *PDNR*, *PN_min_*, and *PNR*, respectively. Inundation frequency, underground biomass, TC, EC, and *PN_min_* effects on N pools accounted for 53%, 69%, and 98% of the variance of NH_4_^+^, NO_3_^−^, and TN, respectively. This suggests that future sea level rise may decrease N storage due to increase in coupled nitrification–denitrification and decrease in N mineralization, and the NH_4_^+^ flux may change from sink to source in intertidal ecosystems.

## 1. Introduction

Intertidal flats constitute major portions of estuaries and are simultaneously some of the most economically important and vulnerable ecosystems on Earth [1,2]. These intertidal flats have particular characteristics, such as being alternately exposed and submerged owing to the periodic ebb and flood tides [1]. These periodic tides usually form an increasing hydrological gradient from upland areas to the shore along the intertidal flats. It is well established that this spatial distribution of hydrological gradients toward the sea reflects not only an increased inundation potential but also a decrease in plant growth and a series of interrelated biotic and abiotic factors [3,4,5]. Nitrogen (N) is a key limiting nutrient in marine and estuarine environments, although N load and incidence of eutrophication increase in the estuaries [6,7,8,9,10]. A key factor mediating the N cycle in intertidal flats is the water regime due to periodic tidewater fluctuations [1]. Furthermore, changing water regimes and succession affects sediment bulk density (BD), salinity, oxidation–reduction condition, pH, organic carbon, nutrient input, sediment delivery, plant growth, etc. [2,11,12,13,14], which may further affect the N cycle.

Global climate change is predicted to affect the water regimes in intertidal flats as a consequence of the heightened occurrence of drought or flooding events [15,16]. Given the changes in hydrological gradients that occur along elevational gradients, estuarine wetlands provide a natural setting for gaining insights into the N cycle under projected climate change scenarios. For two decades, sediment N cycling along hydrological gradients have raised global concerns, including the N pools and processes, and inorganic N fluxes across the sediment–water interface (Table 1); however, previous results have been unclear. For instance, sediment total N (TN), nitrate N (NO_3_^−^), rates of nitrification, denitrification, and mineralization increased as inundation increased [1,14,17,18,19,20,21,22,23,24,25]. Nevertheless, no significant spatial variation in NH_4_^+^ concentration and denitrification rates [23,26,27], and decreasing mineralization rates, TN, NH_4_^+^, and NO_3_^−^ concentration [28,29,30,31] have also been found along an increasing hydrological gradient. Moreover, NH_4_^+^ and NO_3_^−^ fluxes across the sediment–water interface were uncertain because both efflux and uptake can be found [11,28,32,33]. However, since these studies focused on N pools, processes, or fluxes independently, the N pools and processes in the subsurface sediment and the links among N pools, processes, and fluxes along hydrological gradients remain poorly known.

A conceptual scheme for the fate of N pools, processes, and fluxes along hydrological gradients can provide a comprehensive perspective to understand the underlying causal mechanisms in intertidal flats, and thus improve predictions and climate adaptation strategies. Episodic events, such as floods, storms, or droughts, lead to a high amount of nutrients entering subtropical estuaries [34,35], and contributes substantially to severe eutrophication [7]. To better understand the mechanisms that control the fate of N in subtropical estuarine wetlands experiencing climate change, we used a space-for-time substitution method to predict the effects of the alternative scenarios of drought or sea level rise in the Min River estuary. To the best of our knowledge, this is the first study to investigate the links and among N pools, processes, and fluxes along hydrological gradients.

Min River estuary located in the subtropical zone is a typical subtropical estuarine habitat. Periodic hydrological dynamics are characteristic in this estuarine wetland due to the tide–surge interaction intensified by the Taiwan Strait [36]. Simultaneously, this wetland receives high levels of N input because of tidal action and human activities [37]. The present study aimed to (1) investigate the spatial distribution of N pools (TN, NH_4_^+^, and NO_3_^−^), processes (nitrification, denitrification, and mineralization) and the sediment–water interface inorganic N fluxes along a hydrologic gradient; (2) understand the mechanisms underlying N pools’ establishment, transformation, fluxes, and environmental parameters; and (3) propose a conceptual scheme for the fate of N pools, processes, and fluxes along hydrological gradients and predict the implications of long-term N storage in estuarine coastal wetlands under conditions of drought or sea level rise conditions.

## 2. Materials and Methods

### 2.1. Study Site

**Figure 1:**
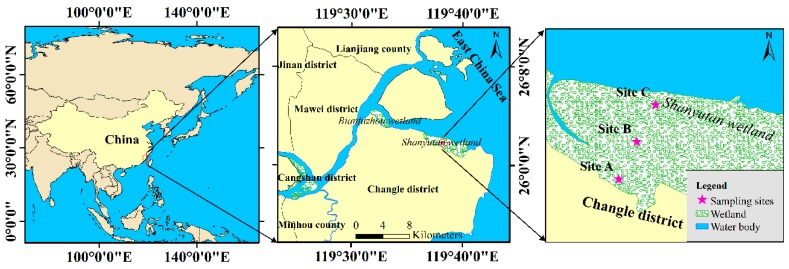
Study area and sampling sites.

The present study was conducted on the Shanyutan tidal marsh of the Min River estuary National Nature Reserve, China (26°0′36″~26°3′42″ N, 119°34′12″~119°40′40″ E) (Figure 1), covering an area of 4.42 km^2^. It represents a typical estuarine habitat of the East China Sea [5]. It is dominated by an East Asian monsoon climate (annual mean temperature: 19.7 °C; annual mean precipitation: 1200–1740 mm) [39]. *Cyperus malaccensis* Lam. var. *brevifolius* Bocklr., *Phragmites australis* (Cav.) Trin. ex Steud., and *Spartina alterniflora* Lois. are the dominant species of vegetation. 

The sampling sites were located at an intertidal flat of Shanyutan tidal marsh, which has a typical hydrological gradient. Three sites, located at 120 m intervals, were sampled: a high tidal flat (site A), an interaction zone between high and middle tidal flats (site B), and a middle tidal flat (site C) (Figure 1). The total inundation periods of sites A, B, and C were approximately 4.15%, 12.06%, and 37.11% of a year, respectively. These sampling sites were all colonized by *C. malaccensis.* More detailed information about the study area can be found in Luo et al. [5] and Zhang et al. [3].

### 2.2. Sampling and Analysis

Sediment samples were collected in August 2013, from sites A, B, and C, during the ebb tide (Figure 1). At each site, three sediment cores (three replicates) were collected using a steel adobe (10 cm diameter; 50 cm depth) and then sectioned into 10 cm samples. All samples were carefully placed in sealed plastic bags, stored in a portable cooler, and transported to the laboratory for analysis. Simultaneously, for later determination of the dissolved inorganic N flux, sediment cores 0–15 cm from the surface were collected from all sites by opaque PVC tubes (30 cm height; 7 cm internal diameter). In a preliminary survey conducted in May 2013, we observed that tidal TN, NH_4_^+^, and NO_3_^−^ were relatively consistent along the creek in the study area; thus, we collected 25 L tidewater from the creek in the same sites for later slurry incubations and sediment–water interface inorganic N flux assay. All samples were transported to the laboratory within 1 h 30 min. The aboveground and underground biomasses were surveyed, and their biomasses were reported on a dry weight mass basis (g dry weight m^−2^).

In the laboratory, the sediment BD was determined using the syringe technique after oven-drying sediment samples at 105 °C until constant weight [39]. Sediment pH was measured with a pH meter (IQScientific Instruments, USA) and electrical conductivity (EC) was measured with a 2265FS EC meter (Spectrum Technologies Inc., USA) using a soil:water ratio of 1:5 [40]. Grain size fractions were determined using laser diffraction (Mastersizer 2000, Malvern Instruments, UK), reported on a volume basis using the Malvern software (version 5.6), and classified as clay (<4 μm), silt (4–63 μm), or sand (63–125 μm) according to the Wentworth scale [41]. Sediment TN and total carbon (TC) were determined using a Vario EL Elemental Analyzer (Elementar, Germany). Sediment NH_4_^+^ and NO_3_^−^ were extracted from 5 g of air-dried sediment with 2 M KCl solution at a liquid:soil ratio of 5:1 [42], and then determined using a flow injection analyzer (Skalar Analytical SAN++, Lachat, The Netherlands). All TC, TN, NH_4_^+^, and NO_3_^−^ values are reported on a dry weight mass basis.

### 2.3. Slurry Incubations

Potential rates of nitrification (PNR)—Assays were performed in duplicate using 10 g of homogenized fresh sediment placed into 250 mL glass incubation bottles, to which 20 mL of in situ tidewater with or without the nitrification inhibitor allylthiourea (10 mg L^–1^) was added. All samples were incubated in the dark for 6 h at the in situ temperature, with stirring (120 rpm). Following incubation, 10 mL of the overlying water was collected for later analysis of ammonia [43]. The NH_4_^+^ and NO_3_^−^ concentrations in the extracted water samples were determined by flow injection analysis.

Potential rates of denitrification (PDNR)—Assays were performed in duplicate using 10 g of homogenized fresh sediment placed into 140 mL glass incubation bottles, to which 50 mL of in situ tidewater was added. The incubation bottles were tightly sealed with silicone rubber stoppers. Samples were assayed in duplicate, either with or without the addition of 20% (v:v) acetylene. A separate set of time-zero samples were assayed immediately after acetylene addition. All samples were incubated in the dark for 6 h at the in situ temperature, with stirring (120 rpm). Prior to the end of the incubation, the oscillator frequency was adjusted to vigorous oscillation for 10 min and 15 mL of the evolved gases was collected from each bottle and stored in 50 mL gas sampling bags [44]. The extracted gas samples were tested using a gas chromatograph (GC-2014, Shimadzu, Japan) to determine the N_2_O concentration.

Potential net soil N mineralization rates (PN_min_)—Assays were performed during 14 d soil incubations. For the laboratory incubation, homogenized fresh sediment (10 g) were placed into 140 mL glass incubation bottles and then preincubated in a dark incubator for 2 days at 25 °C. Bottles were sealed using cling wrap to prevent moisture loss, and water content was checked by weighing and adjusted as needed. After incubation, samples were extracted on days 1 and 14 by using 50 mL of 2 M KCl (shaken at 250 rpm for 1 h [45]. The extracts were filtered (0.45 μm) and then frozen at −20 °C for later analysis of NH_4_^+^ and NO_3_^−^ by flow injection.

The *PNR* (nmol g^−1^ h^−1^) was calculated as the change in an incubated sample with and without the nitrification inhibitor allylthiourea [Equation (1)], while *PDNR* (nmol g^−1^ h^−1^) was calculated as the change in an incubated sample with and without acetylene [Equation (2)]. The *PN_min_* (mg kg^−1^ d^−1^) was calculated after 14 d sediment incubations as the change in NH_4_^+^ and NO_3_^−^ between the initial and incubated samples [Equation (3)].

(1) $$PNR=\;\frac{\Delta N_{1}\;\times \left(20\;+\;10\;\times \;M\right)\;\times \;1000}{T\;\times \;W\;\times \;\left(100\;-\;M\right)}$$

(2) $$PDNR=\;\frac{\Delta N_{2}\;\times \;V\;\times \;1000}{T\;\times \;W\;\times \;\left(100\;-\;M\right)}$$

(3) $$PN_{min}=\;\frac{\Delta N_{3}}{T}$$

where Δ*N_1_* (mmol L^−1^) is the change in NH_4_^+^ concentrations with and without the nitrification inhibitor allylthiourea, Δ*N_2_* (mmol L^−1^) is the change in N_2_O concentrations with and without acetylene, and Δ*N_3_* (mg kg^−1^) is the change in inorganic N (NH_4_^+^ and NO_3_^−^) concentrations before and after incubation; *T* is the incubation time. *W* (g) is the fresh weight, *M* (%) is the moisture content, and *V* (mL) is the air volume of the incubated bottle.

### 2.4. Dissolved Inorganic Nitrogen Flux Measurements

Nine cores (three sampling sites × three replicates) in opaque PVC tubes were installed into a continuous-flow system, placed within a flow-through system. This system was hermetically sealed and opaque and consisted of a square incubation box (height 50 cm; length 20 cm), a carboy to contain the in situ and unfiltered tidewater, a multichannel peristaltic pump, polyether-ether-ketone transmission tubing, and an acetol plunger with a Viton O-Ring (DuPont, Wilmington, USA). Tidewater was poured into the incubation box until the water level was 4 cm higher than that of the PVC tube. The pump transferred water from the carboy to continuously displace the water overlying the core at a flow rate of 4.5 L min^−1^ (through the whole system, including the nine cores). Each core was pre-incubated for 24 h to establish steady-state exchange conditions. Then each core was cultivated at the time of in situ flooding; the inflow and outflow water samples at time 0 and time of the end of cultivation (40 mL) were collected using a syringe (100 mL), respectively, then filtered through 0.45 μm pore size filters for NH_4_^+^ and NO_3_^−^ analysis. The exchange fluxes of NH_4_^+^ and NO_3_^−^ across the sediment–water interface were calculated according to the following equation (Equation (4)) [46].

(4) $$F=\;\frac{V\;\times \;\Delta C}{S\;\times T}$$

where *F* (mmol m^−2^ h^−1^) is the NH_4_^+^ or NO_3_^−^ flux, Δ*C* (mmol L^−1^) is the change of NH_4_^+^ or NO_3_^−^ concentration, *V* (L) is the volume of overlying water running above the surface of the core sediment, *S* (m^2^) is the surface area of the core sediment, and *T* (day) is the incubation time.

### 2.5. Statistical Analyses

Differences among the different sites were analyzed using a one-way analysis of variance (ANOVA) and repeated measure analysis of covariance (ANCOVA) in SPSS 22.0 (SPSS Inc., Chicago, IL, USA) for Windows 10.0 (MI, Beijing, China). All data were tested for normality and homogeneity of variance prior to ANOVA using the Shapiro–Wilk test and the Brown–Forsythe test, respectively. If these assumptions were not met, then the raw data were log transformed before any further statistical analysis. The *F*-values from the main tests and the t-statistic from the pairwise comparisons were evaluated in terms of the significance of the tested factor among different groups at *p* < 0.05. Structural equation modeling (SEM) was performed to analyze the causal mechanisms underlying N pools’ establishment, processes, and environmental parameters. The SEMs were implemented using SPSS Amos 21.0 (IBM, Armonk, New York, NY, USA). The best-fit SEM was derived by maximum likelihood and the model fit was determined using *P*-values, chi-square tests (χ^2^), root mean square errors of approximation (RMSEA), goodness-of-fit index (GFI), and Akaike information criteria [47]. Plots of N pools, processes, and fluxes were generated using Origin 9.3 (OriginLab Corporation, Northampton, MA, USA), and SEM outputs and a conceptual framework were created using Microsoft Office Visio 2016 (Microsoft Corporation, Redmond, Washington, DC, USA).

## 3. Results

### 3.1. Plant Biomass and Sediment Geochemistry along a Hydrological Gradient

We found relatively high sedimentary BD in site B at 0–10 and 20–30 cm, but in site A at 10–20 and 30–50 cm (Table 2). At 0–20 cm, TC concentration (15.37–39.36 mg g^−1^) decreased with elevated inundation frequencies (*p* < 0.05), and there were no significant differences for EC (*p* > 0.05). However, an accumulation of TC and high levels of EC were observed in the subsurface of site B (10–50 cm) (Table 2). Furthermore, sedimentary pH ranged from acidic (pH = 5.88–6.81) to neutral, or slightly alkaline (pH = 7.17–7.66) with elevated inundation frequencies (Table 2). A predominance of silt was observed within the study area (63–75% of all particles). The sand content was significantly lower in the surface sediment of site B than at sites A and C (*p* < 0.05) (Table 2). In the subsurface sediments, higher clay content and lower silt content were observed at site A compared to that at site C (*p* < 0.05) (Table 2). In addition, the biomasses in site A (aboveground: 1188 ± 162 g m^−2^, underground: 1906 ± 196 g m^−2^) were much higher than in site C (aboveground: 888 ± 55 g m^−2^, underground: 586 ± 45 g m^−2^), but similar to that in site B (aboveground: 1008 ± 93 g m^−2^, underground: 1727 ± 280 g m^−2^).

### 3.2. Distribution of Nitrogen Pools, Processes, and Fluxes

**Figure 2:**
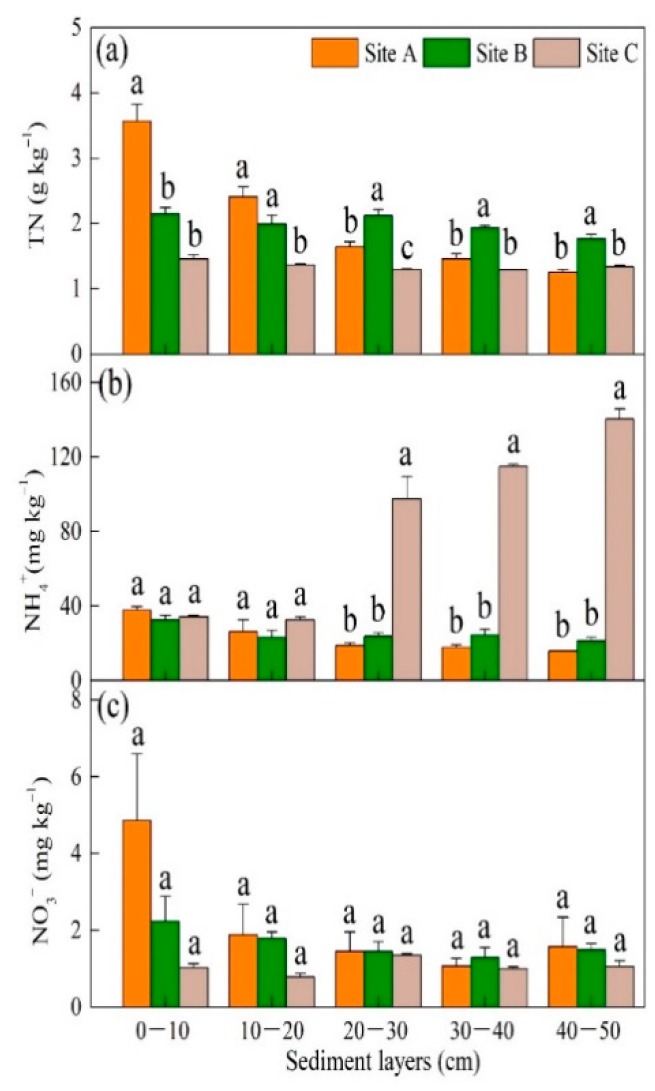
Sedimentary concentrations along the inundation gradient for (**a**) total nitrogen (TN), (**b**) ammonium N (NH_4_^+^), and (**c**) nitrate N (NO_3_^−^) concentrations along the hydrological gradient (mean ± SE). Different letters above bars denote significant difference among sites (*p* < 0.05). Sites A, B, and C refer to the high tidal flat, interaction zone between high and middle tidal flats, and middle tidal flat, respectively.

TN concentration (1.25–3.57 g kg^−1^) decreased with elevated inundation frequencies and decreased elevation except for the accumulation on the subsurface sediment of site B (Figure 2a). Most NH_4_^+^ (15.83–140.15 mg kg^−1^) and NO_3_^−^ (0.78–4.85 mg kg^−1^) concentrations were similar (*p* > 0.05) (Figure 2b,c), but NH_4_^+^ concentration was highest on the subsurface of site C (*p* < 0.05) (Figure 2b).

**Figure 3:**
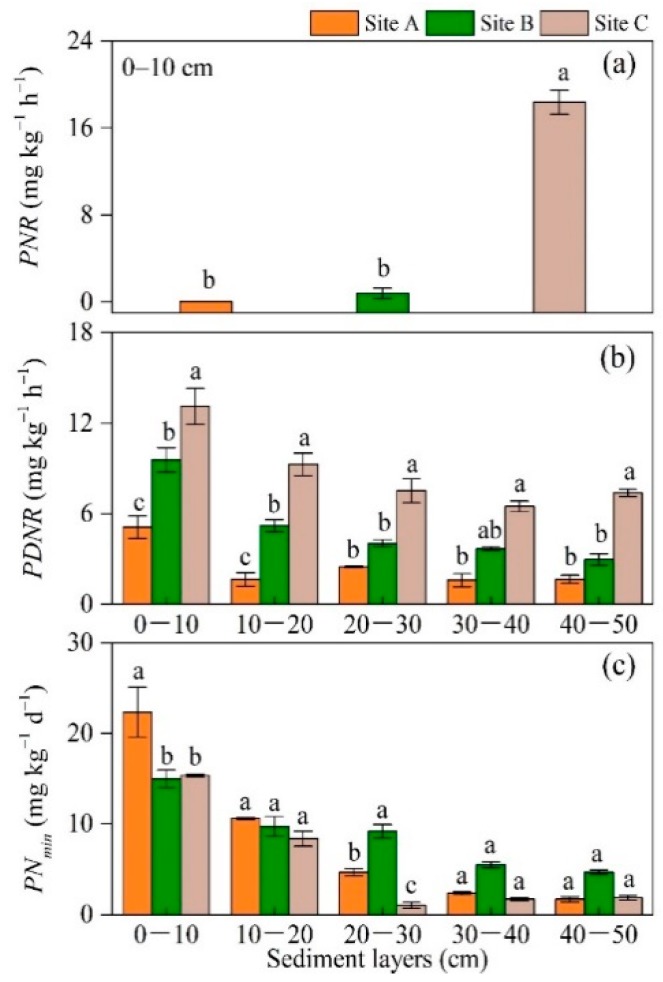
Measured (**a**) potential rates of nitrification (*PNR*), (**b**) potential rates of denitrification (*PDNR*), and (**c**) potential rates of mineralization (*PN_min_*) along the hydrological gradient (mean ± SE). Different letters above bars denote significant differences among sites (*p* < 0.05). Sites A, B, and C refer to the high tidal flat, interaction zone between high and middle tidal flats, and middle tidal flat, respectively.

The *PNR* ranged from 0.03 to 18.38 nmol g^−1^ h^−1^ with a crest value in the surface sediment of site C (Figure 3a), but there was no evidence of *PNR* in the subsurface sediments. The distribution of *PDNR* (0.51–16.17 nmol g^−1^ h^−1^) was relatively consistent, and increased with elevated inundation frequencies and decreased with depth (Figure 3b). The *PN_min_* (1.04–22.34 mg kg^−1^ d^−1^) decreased with depth, and relatively high values were found on the surface sediment of site A and subsurface sediment of site B (*p* < 0.05) (Figure 3c).

**Figure 4:**
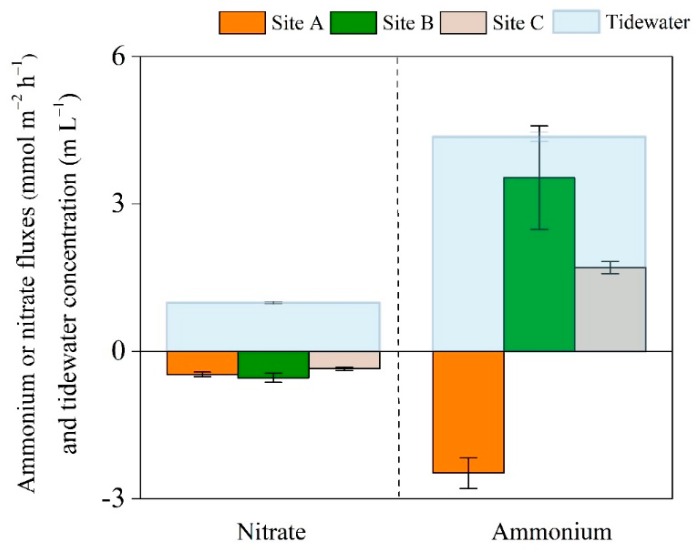
Fluxes of ammonium and nitrate at the sediment–water interface along the hydrological gradient. A positive flux means that ammonium or nitrate are released from the sediment to the overlying water, whereas a negative flux means that ammonium or nitrate are diffused from the overlying water to the sediment. Blue areas represent the ammonium or nitrate concentration of tidewater. Vertical bars represent standard errors (n = 3). Sites A, B, and C refer to the high tidal flat, interaction zone between high and middle tidal flats, and middle tidal flat, respectively.

Exchange fluxes of NH_4_^+^ and NO_3_^−^ across the sediment–water interface are shown in Figure 4. Uptake of NO_3_^−^ was detected at all three zones, varying from −0.54 to −0.35 mmol m^−2^ h^−1^ (Figure 4), whereas NH_4_^+^ fluxes (approximately −2.48 to 3.54 mmol m^−2^ h^−1^) changed from uptake at site A to efflux at sites B and C (Figure 4).

### 3.3. Structural Equation Modeling Analysis of the Drivers, Causal Links, and Contribution to the Priming Effect

**Figure 5:**
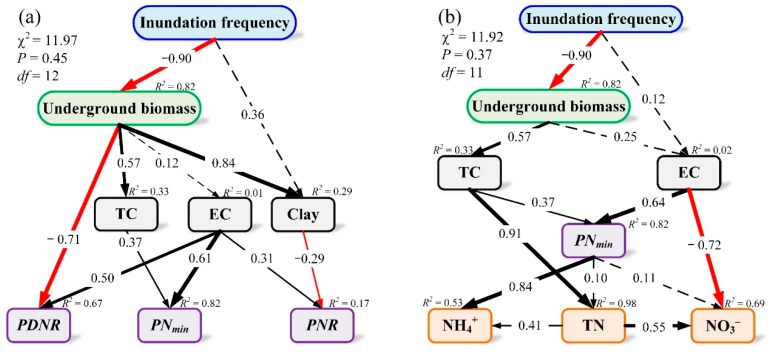
A structural equation model (SEM) used to assess causal mechanisms among N pools, processes, and environmental parameters. (**a**) Effects of inundation frequency, underground biomass, total carbon (TC), electrical conductivity (EC), and clay on N processes (potential rates of nitrification (*PNR*), denitrification (*PDNR*), and mineralization (*PN_min_*)) and (**b**) effects of inundation frequency, underground biomass, TC, EC, and *PN_min_* on N pool (total N (TN), ammonium N (NH_4_^+^), and nitrate N (NO_3_^−^)). The width of arrows indicates the strength of the standardized path coefficient. Black lines indicate positive path coefficients, and red lines indicate negative path coefficients. The solid lines indicate significant (*p* < 0.05), and dashed arrows indicate no significant difference (*p* > 0.05). *R*^2^ values associated with response variables indicate the proportion of variation explained by relationships with other variables.

To quantify the relative importance of the different factors determining N pools and processes, two SEMs were constructed (Figure 5). The SEM of the effects of environmental parameters on N processes and of the coupling of environmental parameters and N processes on N pools showed reasonable fits (χ^2^ = 11.97, *P* = 0.45, GFI = 0.94, RMSEA < 0.001 and χ^2^ = 11.92, *P* = 0.37, GFI = 0.95, RMSEA < 0.05, respectively, Figure 5). The models accounted for 67%, 82%, and 17% in the variance of *PDNR*, *PN_min_*, and *PNR*, respectively (Figure 5a), and for 53%, 69%, and 98% of the variance in NH_4_^+^, NO_3_^−^, and TN, respectively (Figure 5b). We found a direct negative effect of inundation frequency on underground biomass, and this underground biomass had a positive effect on sedimentary TC and a negative effect on *PDNR* (Figure 5a). Furthermore, sedimentary TC has positively correlated to *PN_min_* and TN concentration, and EC was positively correlated to *PDNR*, *PN_min_*, and *PNR* (Figure 5a). In addition, a positive correlation was noted between *PN_min_* and NH_4_^+^, and TN was positively correlated with both NH_4_^+^ and NO_3_^−^ (Figure 5b).

## 4. Discussion

### 4.1. Spatial Distribution of Nitrogen Processes

In the present study, both *PDNR* and *PNR* increased with an elevated hydrologic gradient, corroborating the results of some previous studies [14,19,20,21,32,33], but were contrary to the results of some other previous studies [23,38]. We found no evidence of *PNR* in the subsurface sediments, which suggested that the nitrification process is not ubiquitous throughout the subsurface. Our measurements of *PDNR* were similar to those of Daya Bay, China (1.8–9.2 nmol N g^−1^ h^−1^) [48], but *NR* increased by a factor of 1–16 more than was found in the Yangtze estuary, China (0.02–1.12 nmol g^−1^ h^−1^) [1]. Both *PDNR* and *PNR* increased in a seaward direction, which might be explained by coupled nitrification–denitrification. Nitrification leads the biological oxidation of NH_4_^+^ to NO_2_^−^ followed by the oxidation of NO_2_^−^ to NO_3_^−^, which fuels denitrification by providing an additional source of NO_3_^−^ [49]. The magnitude of coupled denitrification might be greater than the magnitude of direct denitrification and is estimated to support ~60–100% of total denitrification in the coastal zone [32,50,51,52].

The increase in *PDNR* with elevated inundation frequency might be explained by three reasons. First, the increase in inundation may originate an anaerobic environment. Denitrification processes are carried out by denitrifiers, and the predominant heterotrophic microorganisms associated with denitrification are facultative anaerobes under low-oxygen or anaerobic conditions [53]. Second, the SEM indicated a direct negative effect of inundation frequency on underground biomass (Figure 5a), similar to that found in previous studies [4,54]. A decrease in the underground biomass of *C. malaccensis* from the high tidal flat to the middle tidal flat further negatively affected *PDNR* (Table 2; Figure 5a). Root-mediated oxygen supply to the rhizosphere has profound effects on microbial processes in the sediments [55] and denitrifiers are known to follow the patterns of plant diversity and belowground shifts [13]. Third, EC, generally representing salinity, was positively correlated to *PDNR* (Figure 5a). A recent study found that a novel halophilic bacterium capable of heterotrophic nitrification–aerobic denitrification (*Vibrio diabolicus* SF16) and isolated from marine sediments can remove 91.82% of NH_4_^+^ and 99.71% of NO_3_^−^ [56].

Relatively high *PN_min_*, however, was found in the high tidal flat, which does not agree with the previous research in the Yellow River delta, China [23]. The most important factors in N mineralization processes might be sedimentary TC and EC. Previous studies suggested that the decreases in the underground biomass production of *S. alterniflora* negatively affected SOM accumulation as inundation frequency increased [54], and our SEM results support such conclusions (Figure 5a). The increased C availability and EC would also favor N mineralization in the high tidal flat [57]. Indeed, the predominant N fixers were heterotrophic bacteria associated with litter and detritus carbon availability [58]. Mineralization in the deeper strata is almost certainly limited by the supply of labile SOM from above [59].

Furthermore, EC and clay might be two factors influencing the nitrification process, but accounted for only 17% of the variance of *PNR* (Figure 5a). A small increase in soil EC of ~0.51 mS/cm, which can be attributed to an increased flux of electron acceptors, pore water mixing, and flushing of salt, promotes an increase in nitrification rates [44,59]. These results indicated that *PDNR* and *PNR* would decrease but the *PN_min_* would increase under a drought scenario. Conversely, the sea level rise scenario may lead to an increase *in PDNR* and *PNR* but a decrease in *PN_min_*.

### 4.2. Spatial Distribution of Nitrogen Pools

In the present study, TN concentrations decreased with elevated inundation frequencies at the surface, corroborating the results of previous studies (Table 1), although, in some studies, TN values were two- or three-fold higher in estuarine marshes in China (1.4–10.1 g kg^−1^) [17,18,30] and coastal marshes in Germany (2.6–6.8 g kg^−1^) [29]. At the subsurface sediment, an elevated TN concentration was found on the interaction zone between high and middle tidal flats. However, in contrast to earlier findings [28], NH_4_^+^ and NO_3_^−^ concentrations were similar along the hydrological gradient except for the accumulation of NH_4_^+^ concentration on the subsurface sediment of the middle tidal flat. These results suggested that the drought scenario may lead to an increase N storage, whereas N storage would decrease but NH_4_^+^ would increase under the sea level rise scenario.

The SEM results indicated that high TC has contributed to the increase in TN concentration at both surface and subsurface sediment (Table 2; Figure 5b), consistent with the results of Ye et al. [30]. The toposequence along a gradient of inundation frequency represents a chronosequence since the soils have developed associated with TC and TN accumulation [2,29,60]. The most indicative of peat development has the lowest BD, highest SOM, highest mass-based TC, and highest mass-based TN, whereas the least indicative of peat development is the opposite [60].

The SEM results suggested that TN and *PN_min_* were positively correlated to both NH_4_^+^ and NO_3_^−^ (Figure 5b). This was mainly caused by N mineralization, which converts N from organic into inorganic forms, including NH_4_^+^ and NO_3_^−^ immobilization [61]. Thus, NH_4_^+^ concentration accumulated on the subsurface sediment of the middle tidal flat, coupled with high *PN_min_* (Figure 2b). However, this process did not significantly change NO_3_^−^ concentration along the hydrological gradient or NH_4_^+^ at the surface sediment. These results are similar to those reported by Jia et al. [23]. This pattern may be caused by inorganic N production, ionic exchange, and plant uptake. Electrical conductivity directly affected *PN_min_*, *PDNR*, *PNR*, and NO_3_^−^ concentration and indirectly affected NH_4_^+^ concentration (Figure 5b). High salinity could enhance NH_4_^+^ and TN, through a balance between production and remineralization [39,62,63,64], and accelerate ionic displacements, such as NH_4_^+^ exchange and adsorption [11,62,65,66,67]. Additionally, immobilization of plant inorganic N uptake and/or microbial inorganic N immobilization decreased due to increased toxicity and ion stress [39,62,68].

### 4.3. Spatial Distribution of Nitrogen Fluxes

Our measurements of dissolved inorganic N flux were close to those typically reported for coastal zones (NO_3_^−^ flux: approximately −0.8 to 1.8 mmol m^−2^ h^−1^; NH_4_^+^ flux: −0.2 to 0.6 mmol m^−2^ h^−1^) [11,28]. The NH_4_^+^ flux changed from uptake to efflux with elevated inundation frequencies (Figure 4). This result is consistent with the literature that diffusion of NH_4_^+^ from the overlying water to the sediment might change so that it is being released from the sediment to the overlying water instead [69]. NH_4_^+^ flux across the sediment–water interface varied from the sink to source along the elevated inundation frequencies that coincided with the sediment pH from acid soil to slightly alkaline (Table 2). Indeed, pH determined the relative proportion of inorganic N that was nitrified when NH_4_^+^ was sufficiently high [70]. The alkaline sedimentary environment was beneficial to acidic amino acids that were resistant to degradation and dissolution, and NH_4_^+^ might be released into the overlying water in slightly alkaline sediments [71].

The NO_3_^−^ fluxes, however, were similar along the in situ hydrological gradients, and this pattern was similar to the sedimentary NO_3_^−^ concentration. The sediment sink or source function for dissolved inorganic N greatly changed both spatially and temporally due to the complex transportation and transformation of dissolved inorganic N near the sediment–water interface [11,72]. The NO_3_^−^ flux across the sediment–water interface was controlled by NO_3_^−^ concentration in the water column [28]. These results revealed that NH_4_^+^ diffuses from the overlying water into the sediment under the drought scenario, whereas NH_4_^+^ would be released from the sediment into the overlying water under the sea level rise scenario.

### 4.4. Implications, Uncertainties, and Future Study

**Figure 6:**
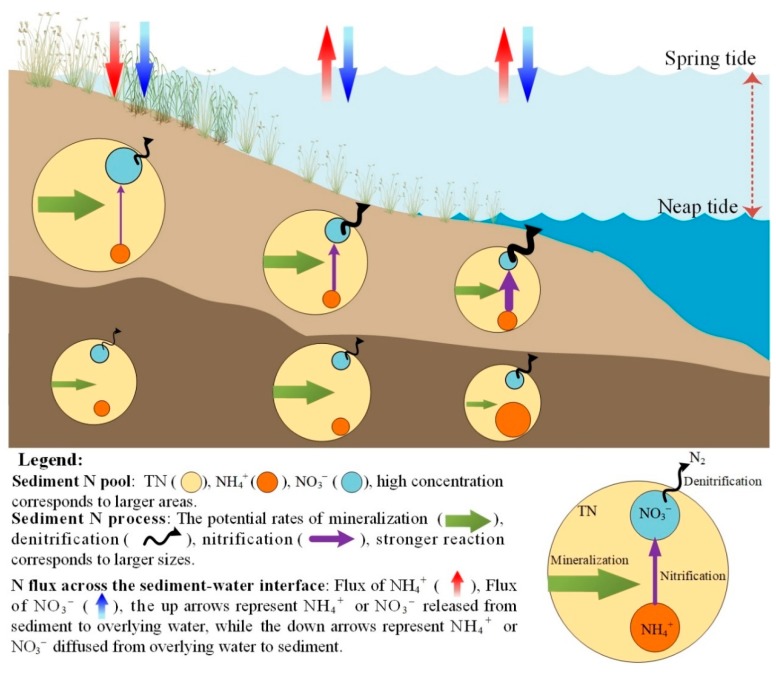
A conceptual schematic for the fate of nitrogen pools, processes, and fluxes in a tidal flat of a coastal wetland ecosystem.

Herein, we proposed a conceptual scheme for the fate of N pools, processes, and fluxes on a tidal flat based on our results (Figure 6). This conceptual scheme can provide a comprehensive perspective to understanding the underlying causal mechanisms in intertidal flats, and it can help improve predictions and climate adaptation strategies. Our data clearly show that *PDNR* and *PNR* increased but *PN_min_* and TN decreased along an elevated hydrological gradient in the surface sediment. However, in the subsurface sediment, we found elevated *PN_min_* and TN concentration in the interaction zone between the high and middle tidal flats, as well as elevated NH_4_^+^ concentration in the middle tidal flat. Additionally, our findings show that N storage may increase owing to increasing drought events. Conversely, under conditions of continuous sea level rise, a decrease in N storage could cause a change from NH_4_^+^ sink to NH_4_^+^ source.

Due to the inherent limitations of the study region, we selected sampling sites that were all covered by *C. malaccensis*. Since plant functional composition is crucial for sediment N pools and processes [17,45], we suggest further investigation of different vegetation-covered areas. We acknowledge that our study lacks a large number of sampling sites, as we sampled only three sites—the high and middle tidal flats and their interaction zone. Further studies with a large number of sampling sites are needed to investigate the N cycle in estuarine wetlands.

## 5. Conclusions

Our data indicated that *PDNR* and *PNR* increased but *PN_min_* and TN decreased along an elevated hydrological gradient in the surface sediment. However, in the subsurface sediment, we found elevated *PN_min_* and TN concentration in the interaction zone between the high and middle tidal flats, as well as elevated NH_4_^+^ concentration in the middle tidal flat. These distribution patterns of N pools and processes may be explained by the decrease in inundation frequency, underground biomass, carbon availability, and the increase in salinity. In addition, NH_4_^+^ flux changed from uptake to efflux with elevated inundation frequencies, which coincided with the change in sediment pH from acid to slightly alkaline soil. However, sedimentary NO_3_^−^ concentrations and NO_3_^−^ fluxes were similar along the hydrological gradients.

In response to possible future scenarios of climate change, N storage and *PN_min_* may increase, but *PDNR* and *PNR* are likely to decrease, and NH_4_^+^ would diffuse from the overlying water into the sediment under a drought scenario. Conversely, the sea level rise scenario may not only lead to an increase in coupled nitrification–denitrification and N removal but also to a decrease in mineralization and immobilization, consequently decreasing N storage and leading to a change from NH_4_^+^ sink to NH_4_^+^ source.

## Figures and Tables

**Table 1:** Nitrogen pool, process, and flux along the gradient of different intertidal flats.

Nitrogen Process		Location	Depth (cm)	Value	Distribution	References
N pool	TN (g kg^−1^)	Yangtze Estuary, China	0–10, 40–100	1.4–7.8	H > M ≈ L	[18]
			10–40		H ≈ M > L	
		Yangtze Estuary, China	0–15	–	H ≥ M ≈ L	[17]
		Petersgroden, Cäciliengroden, and Neβmersiel Germany	0–15	2.6–6.8	H > M > L	[29]
		Nordschweiburg and Dangast, Germany	0–15	3.1–4.2	M > L ≥ H	[29]
		Luoyuan Bay, China	0–40	5.4–10.1	H > M > L	[30]
			40–131		M ≈ L > H	[30]
	NH_4_^+^ (mg kg^−1^)	Tagus Estuary, Portugal	0–5	0.004–4.1	L > H	[28]
		Yellow River Delta, China	0–10	1.0–6.5	H ≈ M ≈ L	[23]
	NO_3_^−^ (mg kg^−1^)	Tagus Estuary, Portugal	0–5	0.03–2.5	L > H	[28]
		Yellow River Delta, China	0–10	0.7–8.4	H ≥ M ≈ L	[23]
N process	*PNR*	East coast of Jutland, Denmark	0–8	–	H > L	[25]
	*PN_min_* (mg kg^−1^ d^−1^)	Yellow River Delta, China	0–10	−0.23–0.24	L ≥ M ≥ H	[23]
	*PDNR* (μmol N m^−2^ h^−1^)	Colne Estuary, UK	0–10	1.1–98.2	H ≈ L	[27]
		Mid-Atlantic Bight, North Atlantic Ocean	0–8	0.006–0.2	W_17m_ > W_15m_ ≈ W_11m_	[32]
		Randers Fjord and Norsminde Fjord, Denmark	0–0.5	–	W_1m_ > W_0.5m_	[33]
		Colne Estuary and Humber Estuary, UK	0–2	0.1–421.7	H > M > L	[38]
		Conwy Estuary, UK	0–2	0–108	M > H > L	[38]
		Weeks Bay Estuarine, USA	0–19	21.6–33.6	M ≈ L	[26]
Inorganic N fluxes at the sediment–water interface.	Flux of NO_3_^−^ (mmol m^−2^ h^−1^)	Tagus Estuary, Portugal	–	−0.8–1.8	H (–), L (+)	[28]
		Mid-Atlantic Bight, North Atlantic Ocean	–	−0.01–0.02	W_11m_ (±), W_15m_ (±), W_17m_ (–)	[32]
		Randers Fjord and Norsminde Fjord, Denmark	–	−120.0–47.0	W_1m_ (–) > W_0.5m_ (–)	[33]
		Yangtze Estuary, China	–	−0.8–0.4	H (±), I (±), M (±)	[11]
	Flux of NH_4_^+^ (mmol m^−2^ h^−1^)	Tagus Estuary, Portugal	–	0.02–0.09	H (+), L (+)	[28]
		Mid-Atlantic Bight, North Atlantic Ocean	–	−0.02–0.12	W_11m_ (±), W_15m_ (+), W_17m_ (+)	[32]
		Yangtze Estuary, China		−0.2–0.6	H (±), I (±), M (±)	[11]

Note: NH_4_^+^, NO_3_^−^, TN, *PNR*, *PDNR*, and *PN*_min_ represent ammonium, nitrate, total nitrogen, nitrification, denitrification, and mineralization, respectively. The flux of NH_4_^+^ and flux of NO_3_^−^ represent flux of ammonium and nitrate at the sediment–water interface. H, M, L, and I represent the high, middle, and low tidal flats and the interaction zone of high and medium tidal flat, respectively. W_xm_ = water deep at x m. “+” represents efflux, “−” represents uptake, and “±” represents alternation of efflux and uptake.

**Table 2:** Bulk density (BD), pH, electrical conductivity (EC), total carbon (TC), and grain size at each site along the in situ hydrological gradients.

Depth (cm)	Sites	BD (g cm^−3^)	pH (1:5, Soil:H_2_O)	EC (mS cm^−1^)	TC (mg g^−1^)	Grain Size (%)		
						Clay	Silt	Sand
0–10	Site A	0.71 ab	5.88 c	3.58 a	39.36 a	26.94 a	68.54 a	4.53 a
	Site B	0.75 a	6.45 b	3.35 a	24.56 b	29.51 a	69.59 a	0.90 b
	Site C	0.63 b	7.32 a	3.29 a	15.70 c	20.40 a	75.28 a	4.32 a
10–20	Site A	0.89 a	6.24 a	2.48 a	27.89 a	34.51 a	63.61 b	1.88 a
	Site B	0.70 b	6.50 a	3.08 a	21.34 b	31.52 ab	66.73 ab	1.75 a
	Site C	0.75 ab	7.17 a	3.04 a	15.37 c	26.08 b	71.12 a	2.81 a
20–30	Site A	0.95 b	6.60 ab	2.13 b	18.49 b	34.98 a	63.55 b	1.46 a
	Site B	0.81 a	6.41 b	2.96 a	22.56 a	34.54 ab	64.18 ab	1.28 a
	Site C	0.85 ab	7.46 a	2.07 b	15.01 c	26.66 b	70.59 a	2.76 a
30–40	Site A	1.01 a	6.81 b	1.89 b	17.74 b	30.98 a	65.99 b	3.04 a
	Site B	0.73 b	6.44 b	2.65 a	20.81 a	32.14 ab	66.48 ab	1.38 a
	Site C	0.84 ab	7.66 a	1.90 b	14.69 c	26.96 b	70.52 a	2.52 a
40–50	Site A	1.03 a	6.58 b	2.00 b	16.29 b	25.92 a	67.01 b	7.08 a
	Site B	0.80 b	6.65 b	2.54 a	20.93 a	27.39 a	69.88 ab	2.73 a
	Site C	0.82 b	7.66 a	2.00 b	14.58 b	23.95 a	72.93 a	3.12 a

Note: Values are means of three replicates. Different letters denote significant differences among sites (*p* < 0.05). Sites A, B, and C refer to the high tidal flat, interaction zone between high and middle tidal flats, and middle tidal flat, respectively.
